# Melioidosis Masquerading as a Mediastinal Abscess

**DOI:** 10.31662/jmaj.2023-0186

**Published:** 2024-06-03

**Authors:** Chee Yik Chang

**Affiliations:** 1 Medical Department, Hospital Sultanah Aminah, Johor, Malaysia

**Keywords:** Melioidosis, splenic abscess, mediastinal abscess

A 57-year-old male farmer with type 2 diabetes mellitus, residing in an area where melioidosis is not endemic, presented with a 3-week history of intermittent fever, accompanied by malaise and weight loss. He denied experiencing chest pain, shortness of breath, or hemoptysis. A chest computed tomography (CT) scan revealed a superior mediastinal abscess and multiple splenic abscesses (Figure 1). The blood culture was positive for *Burkholderia pseudomallei*, confirming the diagnosis of melioidosis, while the tuberculosis test was negative. The bronchoscopy examination revealed no abnormalities. The patient received a 4-week course of intravenous ceftazidime, followed by oral trimethoprim-sulfamethoxazole. A follow-up CT scan revealed that the mediastinal and splenic abscesses had completely resolved.

**Figure 1.**
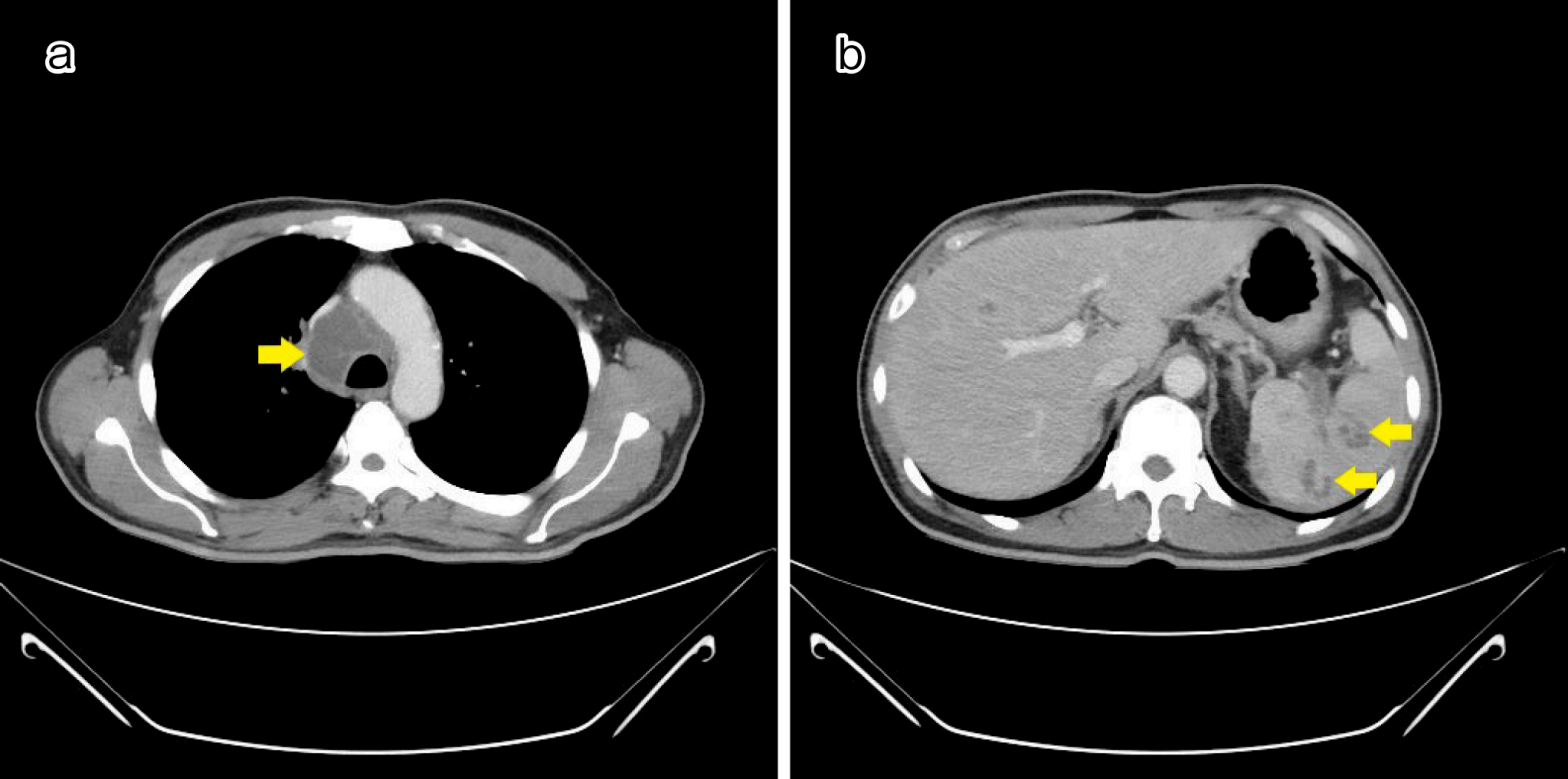
A computed tomography scan showing (a) superior mediastinal abscess and (b) multiple splenic abscesses.

Melioidosis can manifest with diverse clinical presentations, including pneumonia, septic arthritis, encephalomyelitis, and internal organ abscesses ^(1)^. Notably, mediastinal abscesses due to melioidosis are uncommon. The Darwin Prospective Melioidosis study found that mediastinal lymphadenopathy/mass was present in 99 out of 1148 patients (8.6%) with culture-confirmed melioidosis ^(2)^. The presence of mediastinal and splenic abscesses should raise a strong suspicion of melioidosis, especially in endemic areas.

## Article Information

### Conflicts of Interest

None

### Author Contributions

CYC: Conception and design of the study, acquisition of data, drafting the article, final approval of the version to be submitted.

### ORCID iD

Chee Yik Chang: 0000-0002-3104-8168

### Informed Consent

Written consent has been obtained from the patient to publish the information, including the photographs.
